# Chloride-Induced Corrosion Performance of ASR-Contaminated Concrete: Coupled Analysis Using Resistance Variation and NT Build 492 Method

**DOI:** 10.3390/ma19020247

**Published:** 2026-01-08

**Authors:** Tianxing Shi, Shami Nejadi, Harry Far

**Affiliations:** School of Civil and Environmental Engineering, University of Technology Sydney (UTS), Sydney, NSW 2007, Australia

**Keywords:** ASR deterioration, chloride-induced corrosion performance, combined deterioration influence

## Abstract

This study examines how the Alkali–Silica Reaction (ASR) modifies chloride transport and chloride-induced corrosion (CIC) in reinforced concrete beams. Non-reactive and reactive concrete beams were cast with blue metal and dacite aggregates and subjected to a two-stage exposure: (i) alkali-rich immersion at 38 °C to induce ASR, and (ii) impressed-current CIC and NT BUILD 492 chloride migration testing. Microstructural changes were characterized using SEM–EDS and TGA. The reactive specimens developed extensive surface cracking, but after one year of ASR exposure, exhibited 47–53% lower non-steady-state migration coefficients (Dnssm: 7.03–8.02 × 10^−12^ m^2^/s) than the non-reactive beam (15.09 × 10^−12^ m^2^/s). After two years, Dnssm was reduced by approximately 37–56% (4.78–6.93 vs. 10.92 × 10^−12^ m^2^/s). Crack mapping confirmed higher crack density and width in reactive beams, while SEM–EDS and TGA evidenced Ca depletion and the formation of C–(N,K)–S–H gels, which fill cracks and refine the pore structure. Electrical resistance monitoring showed earlier corrosion initiation in ASR-damaged beams but less pronounced resistance loss during the propagation phase. Overall, the results indicate that ASR can initially accelerate corrosion initiation through microcracking and reduced resistivity, but long-term gel deposition can partially seal transport paths and lower chloride migration under the specific conditions of this study.

## 1. Introduction

The Alkali-Silica Reaction (ASR) is a complex mechanism of alkalis, such as sodium oxide and potassium oxide from cement, and silica from amorphous silicate aggregates [1,2]. This reaction produces the ASR gel, causing expansion inside the reactive aggregate and cracking the aggregate [3,4]. The aggregate cracks further expand the aggregate volume, destroy porous structures, cause matrix cracks on the concrete structure surface, and decrease the structure’s design strength and durability [5]. Meanwhile, chloride-induced corrosion (CIC) is another deleterious factor that affects reinforcing concrete structures [6,7]. The general chloride attack problems share similar aggressive environmental conditions with the ASR problem, such as high-humidity regions and structures exposed to an aggressive environment with high concentrations of sodium, chloride, and hydroxide compounds [5]. The chloride-induced corrosion (CIC) was initiated by the chloride ion penetrating the concrete cover and electrochemical reactions, where iron is oxidized to ferrous ions, which then react with hydroxide ions to form corrosion products such as FeCl_3_, Fe(OH)_2_, and Fe(OH)_3_ [8]. These products expand in volume, causing internal pressure, which can lead to cracking and concrete spalling.

The combined deterioration of ASR and CIC in concrete structures has been reported several times, and they are all related to coastal region infrastructures. Generally, the lifespan of infrastructure is designed for over 50 to 100 years of service. After two years of service, the Lucinda Jetty in Queensland, Australia, reported severe deterioration due to ASR and chloride-induced corrosion [5]. Moreover, the annual reports of the American Federal Highway Administration and the BRE Centre for Concrete Construction showed that the expenses and losses due to ASR and chloride-induced corrosion were $5 billion USD and £750 million GBP, respectively [9,10]. The investigation reports of those cases all highlight the ASR cracks, which cause further chloride-induced corrosion on the reinforcement. It is generally acknowledged that the ASR can accelerate and exacerbate chloride-induced corrosion [5,9,10].

According to Trejo et al. [11], previous research on the combined effects of ASR and chloride-induced corrosion (CIC) indicates that internal cracks generated by ASR significantly impact the initiation and propagation of chloride-induced corrosion by providing alternative transport pathways. The microcracking due to ASR compromises the integrity of the concrete matrix and the bonding between cementitious materials and reinforcement [11]. In later research by Mangat et al. [12], ASR-induced cracks, particularly in the interfacial transition zone (ITZ), facilitated the penetration of chloride ions toward the reinforcement, thereby accelerating the deterioration process and promoting corrosion initiation. However, the combination of ASR and chloride-induced corrosion is a complex phenomenon, according to Mazarei et al. [13]. Previous works on the synergy between ASR and CIC have shown that ASR gel propagating in microcracks can partially fill the pores and fissures, potentially blocking chloride transport locally. Nevertheless, the dissolution of silica and the formation of ASR gel in the reinforcement and cement-bonding region result in a pH value reduction in the pore solution, lower than 12 [14]. Reducing the pH around the reinforcement area lowers the critical chloride threshold and facilitates earlier corrosion [15]. Trejo et al.’s [11] research illustrated that the ASR gel resulted in a dual deterioration mechanism, in addition to microcracking, which reduced the pH value. This theory was also revealed by Barragan-Ramos et al. [15], which evidenced that ASR gel influences the local electrochemical environment, specifically by reducing the pH at the steel’s surface. Recent multiscale investigations of nano-silica-modified geopolymers have likewise demonstrated that gel-network homogeneity and nano-scale refinement of the pore structure strongly influence both mechanical response and chloride transport capacity, underscoring the central role of microstructure in durability performance [16].

The combination of the alkali-silica reaction (ASR) and chloride-induced corrosion (CIC) in concrete structures affects various aspects, ranging from mechanical properties to chemical degradation. The mechanical properties are influenced by microcracks generated by expansive ASR gel [17]. The microcracks damaged the bonding between concrete and reinforcement, compromising structural integrity, and provided additional pathways for chloride penetration, resulting in a reduction in corrosion time [11]. For chemical degradation, the ASR reaction process alters the pore solution pH, which affects the chloride threshold for steel de-passivation [18]. Nevertheless, after propagation of the ASR gel, the products of ASR temporarily fill the microcracks, thereby influencing the ingress and diffusion rate of chloride ions in a complex and nonlinear manner [19]. These conflicting performances and interruptions between ASR and CIC indicate a complex relationship.

To clarify the complex interaction between ASR and chloride-induced corrosion (CIC) in reinforced concrete, this study aims to assess their combined impact on the microstructure and electrochemical performance of the concrete specimens. The samples were manufactured using non-reactive and reactive aggregates for ASR, and non-reactive samples were considered a reference group for CIC performance. The ASR acceleration method is a modified macroscale method derived from previous studies using internal and external alkali equilibrium, together with constant temperature and humidity [20,21]. Each specimen was reinforced with a wire to accelerate CIC’s diffusion using a DC power supply. This method partially immerses the specimens at 50% of their height in a 3% NaCl solution [22,23]. A digital data acquisition system continuously monitored voltage and current during the accelerated electrochemical process. This method enables the quantitative evaluation of time-dependent resistance changes that correlate with corrosion kinetics [24,25,26].

Previous experimental and field investigations have reported that ASR can exert competing influences on chloride transport and corrosion, whereby microcracking tends to accelerate ingress, while gel formation and local densification may, over time, partially restrict transport pathways [11,19]. These studies, however, have primarily focused on small-scale specimens or have examined transport and microstructure separately, leaving a gap in quantitatively resolving how this dual behaviour manifests at the structural scale under a realistic, long-term exposure regime. In the present work, the two-stage influence of ASR on chloride migration is therefore not proposed as a fundamentally new conceptual mechanism, but is instead examined as a quantitatively characterized, time-dependent response in large reinforced concrete beams subjected to a macro-scale alkali-equilibrium regime.

The progression of ASR in microstructure was characterized using scanning electron microscopy coupled with energy dispersive X-ray spectroscopy (SEM-EDS) [27,28] and thermogravimetric analysis (TGA) [29,30]. The SEM-EDS method is a common microanalytical approach for ASR products in concrete structures, providing morphological and compositional analysis [31]. The critical elements evaluated in SEM-EDS for ASR primarily target silicon (Si), calcium (Ca), sodium (Na), and potassium (K) [32]. The TGA was used to quantify compositional variations in cementitious materials by assessing mass differences as a function of temperature [28,31]. For ASR product detection, the typical temperature range is from ambient (25 °C) to 1000 °C [27,33]. During exposure, these test methods facilitated detailed observation of ASR gel morphology, critical chemical concentrations, and hydration product transformations. Additionally, the corrosion rate of the reinforced concrete structures was assessed using a combination of electrical resistance differential measurements, which reflect changes during the exposure period, and steel corrosion progression [25,34]. The rapid migration test method (NT BUILD 492) [35] was conducted to quantify the chloride migration coefficient and evaluate chloride transport capacity and corrosion susceptibility [36,37,38].

## 2. Materials and Exposure Conditions

### 2.1. Raw Materials

The material preparation and specimen manufacturing processes for this study were specifically designed to replicate the deterioration of the alkali–silica reaction (ASR) and subsequent chloride-induced corrosion in reinforced concrete. Two concrete groups were prepared to achieve a controlled evaluation of deterioration mechanisms: a reference group using non-reactive aggregate and a test group using reactive aggregate. The reference group samples utilized non-reactive blue metal aggregate with average particle diameters of 10 mm and 20 mm, complying with Australian Standard 2758.1 [39]. The blue metal aggregate showed negligible ASR expansion in previous research, according to the accelerated mortar bar test (AMBT, ASTM C1260) [40,41].

**Figure 1:**
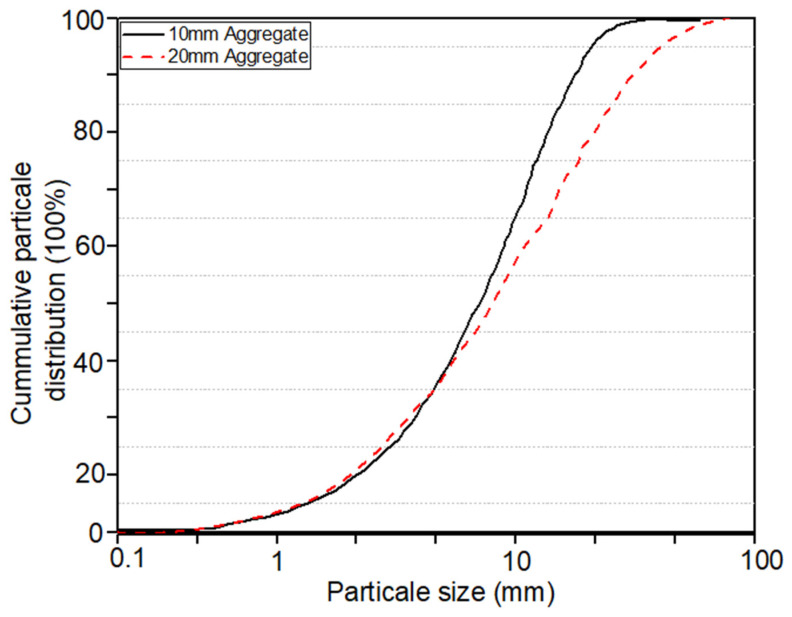
Particle size distribution of the raw 10 mm and 20 mm non-reactive and reactive aggregates used in this study.

**Figure 2:**
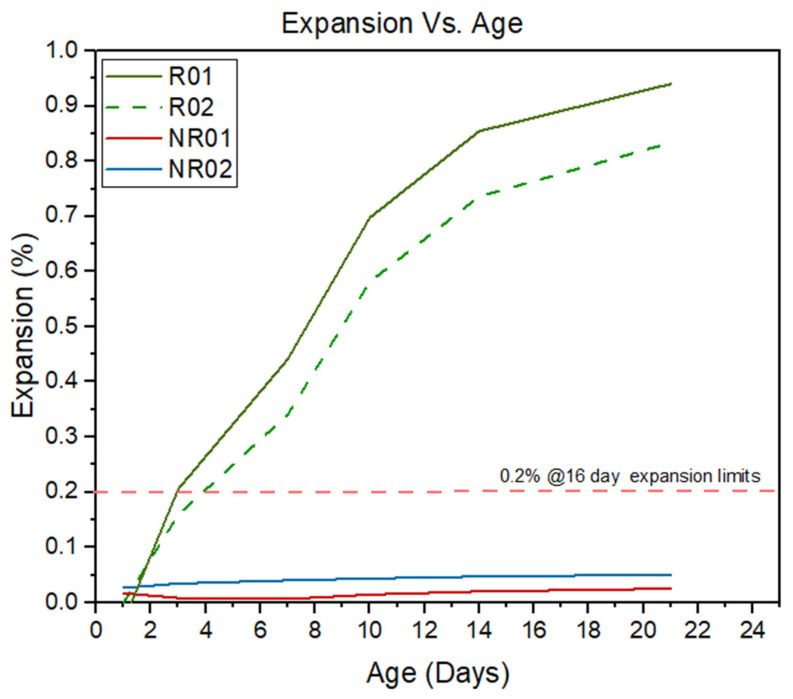
AMBT results of selected aggregates.

The aggregate blend consisted of 70% of 20 mm particles and 30% of 10 mm particles, matching conventional field grading (Figure 1). Furthermore, the test specimens were prepared using reactive dacite coarse aggregate sourced from Hanson’s Canberra quarry (Williamsdale, NSW, Australia). According to previous research, the Accelerated Mortar Bar Test (AMBT), conducted in accordance with ASTM C1260 [40] and AS 1141.60.2 [42], was used to evaluate the susceptibility of the selected aggregates to alkali-silica reaction (ASR) (Figure 2). Mortar bars prepared with the test aggregates were immersed in 1 M NaOH at 80 °C, with expansions monitored over 21 days. The standard expansion threshold of 0.2% at or beyond 16 days was used as the criterion for identifying deleterious reactivity. The results confirmed that the reactive dacite aggregates (R01 and R02) exhibited substantial expansion, both exceeding the 0.2% limit early in the exposure period and reaching close to 0.90% by day 21, thereby classifying them as highly reactive. In contrast, the non-reactive blue metal aggregates (NR01 and NR02) showed negligible expansion, consistently below the deleterious threshold, validating their role as a suitable reference group. These findings, supported by suppliers’ data and prior research, demonstrate that dacite aggregates are highly reactive and appropriate for ASR deterioration studies, while blue metal aggregates serve effectively as stable controls. The same particle size distribution—70% 20 mm and 30% 10 mm was applied to ensure consistent mix composition between reference and test specimens.

General-purpose (GP) cement from Boral conforms to AS 3972 [43]. The cementitious material was used as the sole binder for all specimens. As presented in Table 1, the chemical composition includes 49.24% CaO, 14.15% SiO_2_, and 0.74% K_2_O. The sodium oxide equivalent (Na_2_Oeq) was calculated to be 0.42%, based on the 0.12% Na_2_O and 0.45% K_2_O contents (Table 1), as further calculated by ASTM C496 [44].

### 2.2. Beam Sample Fabrication

The concrete mix designs include reactive aggregate (dacite) and non-reactive aggregate (Blue metal). The mixed proportions of the two concretes are presented in Table 2.

**Figure 3:**
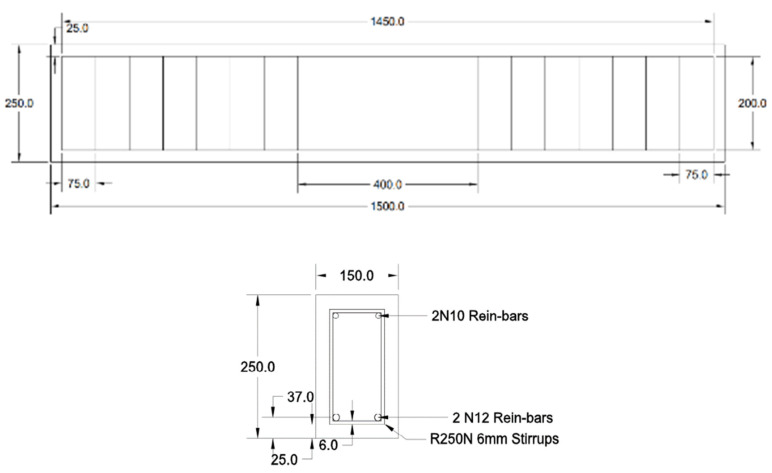
CAD drawing of the sample with reinforcement layouts.

The specimens used in this experiment are large-scale reinforced concrete beam samples with dimensions of 1500 mm × 150 mm × 250 mm (Figure 3), specifically designed to investigate the influence of the alkali–silica reaction (ASR) and chloride-induced corrosion (CIC) under simulated environmental exposure. The 28-day compressive strength testing, conducted with concrete cylinders, showed that the non-reactive and reactive mix achieved an average strength of 45 MPa. The sample arrangement have shown in Table 3.

### 2.3. Exposure Method and Condition

This study adopts a 2-stage exposure methodology to simulate the sequential development of Alkali–Silica Reaction (ASR) and Chloride-Induced Corrosion (CIC) in reinforced concrete. The ASR exposure process initiates with an alkali equilibrium environment to replicate ASR in reactive aggregate specimens. The method is modified to replicate ASR progression in macroscale reinforced concrete samples based on previous research by Thomas et al. [1,20]. The internal alkali content of all reactive specimens was boosted to 1.1% Na_2_Oeq, following performance-based alkali-equilibrium protocols proposed by Thomas and co-workers [20,45]. This value represents an upper-bound, yet field-relevant alkali level for high-alkali concretes, chosen to ensure the development of significant ASR damage in the highly reactive dacite aggregate while avoiding unrealistic, excessively aggressive conditions. At the same time, the external NaOH concentration is matched using Equation (1), producing an external solution concentration of 0.77 mol/L, Equation (2). For the ASR stage, a constant temperature of 38 ± 2 °C was adopted following macro-scale alkali-equilibrium ASR performance tests [20,45], which deliberately use moderate temperatures to accelerate ASR while limiting thermally induced microcracking and microstructural artefacts that may arise at 60–80 °C in large concrete elements.

The ASR acceleration regime was designed following an internal–external alkali equilibrium concept to minimize uncontrolled alkali depletion during long-term immersion. The internal alkali content of the concrete was first increased to 1.1% Na_2_Oeq through cement alkali contribution and additional NaOH, and the external solution was then prepared at the corresponding equilibrium concentration to maintain a high pH around the specimen surface throughout the exposure period. Under these conditions, any alkali leaching from the pore solution into the reservoir is counterbalanced, in first approximation, by the elevated alkali content of the surrounding solution, thereby limiting net alkali loss from the concrete. Although some redistribution of alkalis between paste, aggregate, and solution is inevitable over the extended exposure durations adopted in this study, all beams—reactive and non-reactive—were immersed in the same solution volume and experienced identical temperature and boundary conditions. This approach ensures that any differences in ASR development and subsequent chloride transport arise primarily from aggregate reactivity rather than from systematic differences in alkali availability.

Equation (1): Alkali equilibrium calculation [1,20,45].

(1) $${NaOH}_{con}\left(\frac{g}{L}\right)={Na}_{2}O_{e\;Cement}\left(\%\right)\times 0.7\;\left(\frac{\frac{mol}{L}}{\%{Na}_{2}O_{e\;}\;}\right)\times 17\;\left(\frac{g}{mol}\;OH\right)\times \frac{40(NaOH\frac{g}{mol})}{17(OH\frac{g}{mol})}$$

Equation (2): Concentration calculation.

(2) $${NaOH}_{con}\;\left(\frac{mol}{L}\right)=\frac{{NaOH}_{con}\left(mol\right)}{solution\;(L)}$$

**Figure 4:**
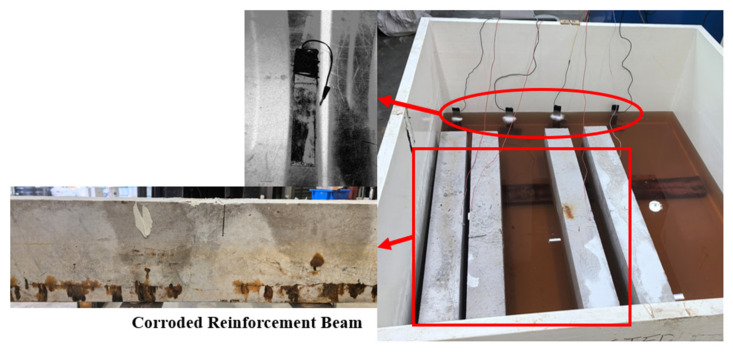
Electrochemical setup for reinforced beam and corroded RC beam.

Following ASR exposure, selected reinforced specimens (NRB1, 2; RB1, 2) were subjected to chloride-induced corrosion under electrochemically accelerated conditions. To replicate high-chloride concentration environments, specimens were partially immersed to 50% of their height in a 3% NaCl solution [24,46]. This exposure level ensures a defined wetting region while leaving the upper portion exposed to air, which is crucial for maintaining oxygen availability for the electrochemical corrosion process. Moreover, an impressed direct current (DC) method was applied to initiate and accelerate steel corrosion [25,47]. Each embedded steel reinforcement was wired externally and functioned as the anode, while an external stainless-steel plate immersed in the same NaCl solution acted as the cathode [48,49]. A constant voltage of 10 V was applied across the circuit, with voltage and current data continuously logged using an integrated data acquisition system. The electrochemical setup of reinforced beam samples is shown in Figure 4.

## 3. Test Methodology

### 3.1. ASR Product Investigation

#### 3.1.1. Scanning Electron Microscopy (SEM-EDS)

This study conducted SEM-EDS on ASR-exposed specimens to investigate internal cross-sections and silica dissolution. The test samples were coated with a metallic layer of gold (Au) or iridium (Ir) of the thickness to 8–12 μm [28]. The SEM image enables observation of morphological features of ASR contamination, such as internal cracks generated from ASR gel propagation and reaction rims at the interface between aggregates and the surrounding cement matrix [50,51]. The imaging was performed at an accelerating voltage of 10 kV and a working distance of 10 mm [52]. Coupled EDS analysis determined the localized element content of ASR products and selected elements. The ASR product element focusing on EDS analysis includes oxygen (O), hydrogen (H), silicon (Si), sodium (Na), calcium (Ca), and potassium (K) [52,53]. This study’s EDS analysis specifically monitors changes in silicon, calcium, and sodium/potassium concentrations in ternary phase diagrams. Therefore, this method revealed alkali migration from the pore solution into reactive silica phases, leading to the formation of ASR gel typically categorized as calcium–sodium–silicate–hydrate (C–N–S–H) or calcium potassium–silicate–hydrate (C–K–S–H) [54,55].

#### 3.1.2. Thermogravimetric Analysis (TGA)

Thermogravimetric analysis (TGA) provides critical insights into the decomposition of reaction products under high-temperature sintering, supporting the SEM-EDS analysis, such as calcium hydroxide (Ca(OH)_2_), calcium silicate hydrate (C-S-H), and ASR gel [29]. The 9-month exposure samples were tested with particles of less than 75 µm in size to ensure homogeneity. A sample mass of 35 mg is subjected to a controlled heating programme, where the temperature increases at a constant rate of 10 °C/min under an inert nitrogen atmosphere with a gas flow of 50 mL/min [29,56]. The temperature range is from ambient to 1000 °C [57]. Critical thermal events are carefully monitored in specific temperature intervals, including 100–200 °C for the loss of non-evaporable water from hydrated phases, 450–600 °C for the decomposition of calcium hydroxide (portlandite), and 600–850 °C for the thermal breakdown of carbonate phases, such as calcium carbonate (CaCO_3_) [56,57]. TGA measures the amount of portlandite and bound and free water, reflecting the degree of hydration and chemical reactivity in the system. These TGA results complement SEM-EDS findings by confirming calcium depletion near reactive silica regions and supporting elemental shifts associated with ASR gel formation [57,58]. Additionally, TGA helps distinguish between expansive ASR gels and beneficial C–S–H phases, which may appear morphologically similar in SEM but differ in thermal decomposition profiles [29,56].

### 3.2. Chloride-Induced Corrosion Evaluation

#### 3.2.1. Time-Dependent Resistance Variation

The electrochemical acceleration method reproduces the CIC deterioration of reinforced concrete samples. Time-dependent resistance is a continuously monitored parameter for chloride ion penetration [48,59]. Regarding previous research on the influence of CIC, the resistance of intact concrete structures remains stable at the early stage of the electrochemical process. It exhibits a slightly decreasing trend, attributed to the saturation of the pore solution and the penetration of chloride ions into the concrete cover [23]. Moreover, as the exposure duration increases, ionic mobility penetrating through the concrete cover initiates passive film degradation, resulting in resistance variation [26,34]. The resistance will exhibit an increasing trend from this stage, attributed to the formation of CIC products such as Fe(OH)_3_ and Fe_2_O_3_·nH_2_O [60,61]. The overall electrochemical acceleration was applied with a constant DC voltage of 10 V. For this study, specimens were partially immersed in a 3% NaCl solution for the entire exposure period. This resistance evolution provides a non-destructive and real-time indicator of corrosion progression, correlating well with the intensity of electrochemical reactions and physical damage. Furthermore, the chloride diffusion resistance was conducted by the test protocol NT BUILD 492 [37,46].

While the impressed-current technique offers a reliable and accelerated approach for studying corrosion initiation and propagation in reinforced concrete, it is important to recognize its inherent limitations. The application of a constant direct current (DC) potential—10 V in this study—can generate elevated current densities that exceed those encountered under natural environmental exposure. As a result, corrosion products may form more rapidly and with different spatial characteristics compared to long-term field conditions. The imposed electrochemical field may also distort the natural transport gradients of chlorides, hydroxides, and other ions, potentially exaggerating steel depassivation rates or producing bar-parallel cracking that differs in morphology from naturally initiated corrosion. Nonetheless, the primary objective of this study was to assess the comparative effect of ASR-induced damage on corrosion performance, and the impressed-current setup provides a consistent and controlled framework for that purpose. All specimens were subjected to identical electrochemical boundary conditions, ensuring that differences in corrosion onset and resistance loss can be attributed to prior ASR effects rather than test variability.

#### 3.2.2. Rapid Chloride Migration Test (NT BUILD 492)

The NT BUILD 492 test [34] was employed to further characterize the chloride-induced corrosion (CIC) performance on ASR-contaminated specimens, assessing the resistance of concrete to chloride ingress [35]. This test method can evaluate chloride transport behaviour, which further supports the resistance variation from electrochemical monitoring [37,46]. The test specimens were exposed to a 10% NaCl solution and a 0.3 N NaOH solution with a voltage of 10–60 V DC [62]. Based on Fick’s second law of diffusion, the NT Build 492 test is an empirical test that details the time-dependent transport of ions in materials with porous structures [63]. Fick’s second law of diffusion was expressed in Equation (3), where C (x, t) is the chloride concentration at depth (x) and time (t), C_s_ is the surface chloride concentration, C_i_ is the initial chloride content, and D_e_ is the effective diffusion coefficient. Nevertheless, the NT BUILD 492 method is further modified based on Fick’s second law, and the non-steady-state migration coefficient ($D_{nssm}$) is calculated by Equation (4) as follows:

Equation (3): Fick’s second law of diffusion to calculate chloride concentration [63].

(3) $$C\left(x,t\right)=C_{s}-(C_{s}-C_{i})\cdot erf(\frac{x}{\sqrt{4D_{e}t}})$$

Equation (4): NT BUILD 492 non-steady-state migration D_nssm_ coefficient calculation [34].

(4) $$D_{nssm}=\frac{0.0236\left(273+T\right)L}{\left(U-2\right)t}(x_{d}-0.0238\sqrt{\frac{\left(273+T\right)Lx_{d}}{U-2}})$$

This study used drilled core samples and standard cylinder specimens (100 mm diameter × 200 mm height). Each sample was sectioned to a thickness of 50 plus or minus 2 mm using a precision cutting machine, with slices extracted from the centre of the drilled core and cylinder, ensuring uniformity in test geometry as recommended by NT BUILD 492 [64,65]. After preconditioning and vacuum saturation, each disc specimen was mounted in the migration test cell, where the applied DC field drove chloride transport over a defined test duration. Upon completion, the specimens were split axially and sprayed with a 0.1 M AgNO_3_ solution to reveal the chloride penetration depth for further calculation [35].

## 4. Result Analyses of the Experimental Programme

### 4.1. ASR Gel Formation by SEM-EDS

**Figure 5:**
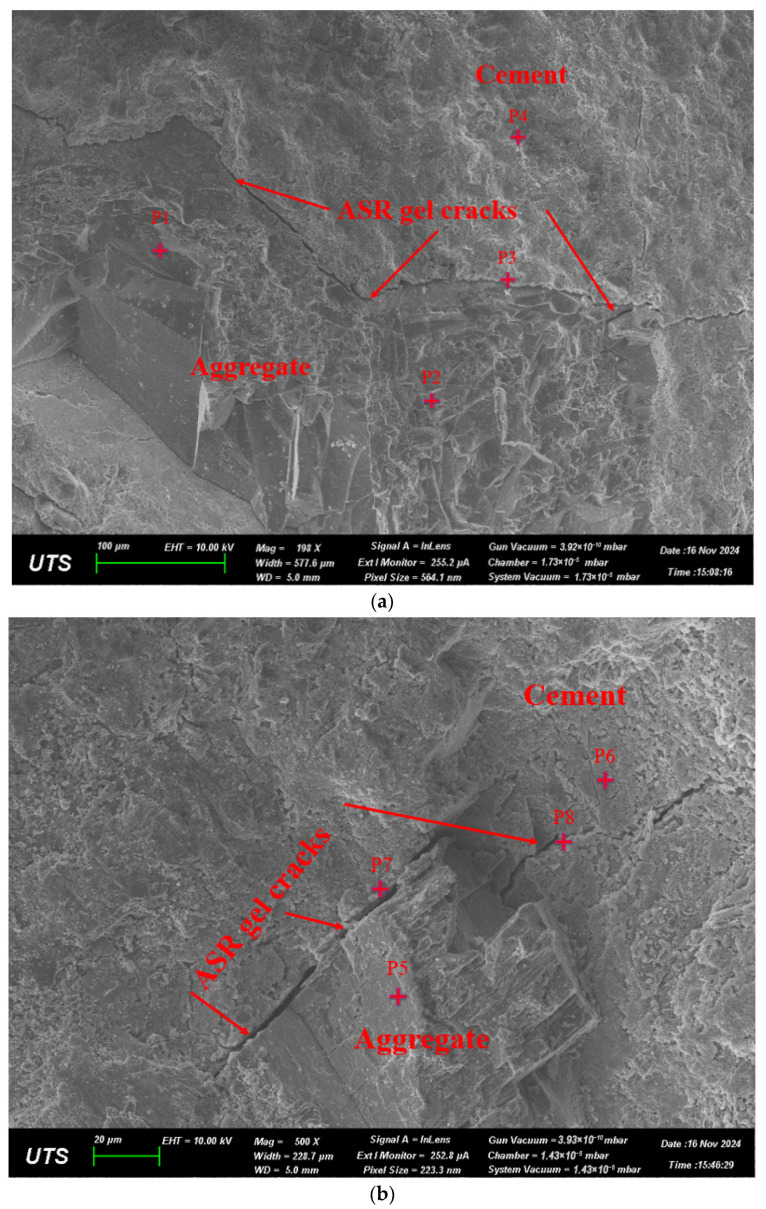
(**a**,**b**): SEM image of 9 M exposed sample. (accelerating voltage 10 kV, working distance 10 mm, scale bar = 50 µm).

**Figure 6:**
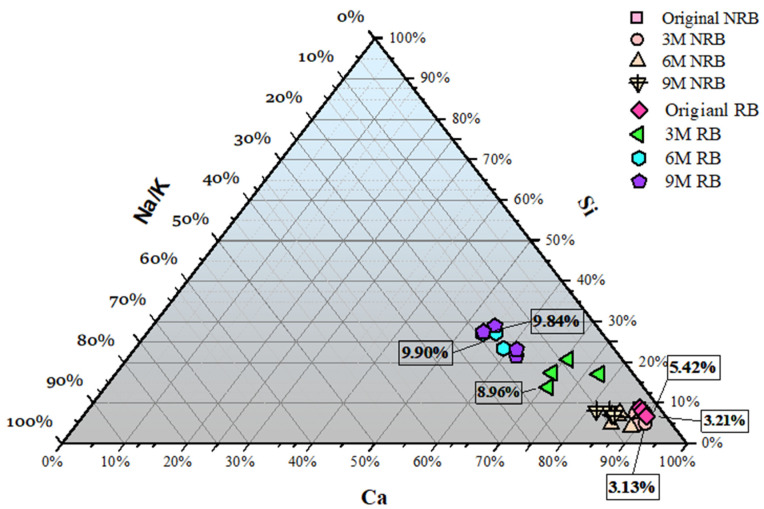
Ternary element variation for 9-month exposure samples.

The SEM micrograph in Figure 5a,b shows a fractured transition zone where cracks propagate from within the aggregate into the adjacent cement matrix for 9-month-exposed reactive RC beams without chloride corrosion. Correspondingly, Figure 6 demonstrates the chemical content comparison of ASR 9-month-exposed beam samples. The labels P1 to P4 in the micrograph correspond to targeted EDS points selected to capture compositional transitions between the aggregate interior, the ASR gel-affected zone, and the surrounding cement paste. The P1 exhibits a silica-depleted, high-calcium signature (Ca 20.7 wt%, Si 5.2 wt%) with elevated sodium, indicating a more advanced reaction rim affected by alkali leaching or silica exhaustion [32,52]. The P2, located near the aggregate–gel interface, indicates a silica-rich and moderately calcium-bearing ASR gel (Si 21.2 wt%, Ca 14.0 wt%) with minor alkalis and aluminum, consistent with partially evolved C-N-S-H gel [52,53]. P3 presents a more chemically balanced gel (Si 16.5 wt%, Ca 12.7 wt%, Na + K 2.0 wt%), likely representing active ASR gel in microcracks, with a Ca/Si ratio (0.77) indicative of moderate expansion potential [50]. Finally, P4, located deeper in the matrix, exhibits increased calcium (24.4 wt%) and reduced silica (7.8 wt%), indicating an interaction of calcium migration, resulting in a Ca-rich modification zone [52].

In Figure 5b, gel-filled cracks extending from the aggregate into the matrix, as marked at points P7 and P8, illustrate the expansive pressure associated with ASR product formation [32,50]. These cracks align along the aggregate–paste boundary and propagate outward, typical of gel-induced fracturing caused by internal swelling pressures. P7, 8, located within the ASR gel cracks, show high silicon (37.4–40.2 wt%) and elevated alkali contents, with sodium and potassium each around 6–7 wt%, and low calcium (4.4–6.6 wt%). These values confirm the presence of alkali-rich, low-calcium ASR gel, which is highly expansive and indicative of active reaction zones. In contrast, P5 presents higher silica (30.3 wt%) and lower alkalis (Na, 0.3 wt%; K, 0.7 wt%), with minimal calcium (2.5 wt%), illustrating an alkali-leached area. P6 shifts to calcium-rich chemistry (Ca 20.9 wt%, Si 10.2 wt%, Na + K 3.1 wt%), representing an altered gel near the cement matrix. The chemical content of selected points is further summarized in Table 4.

### 4.2. TGA/DTG Analysis

**Figure 7:**
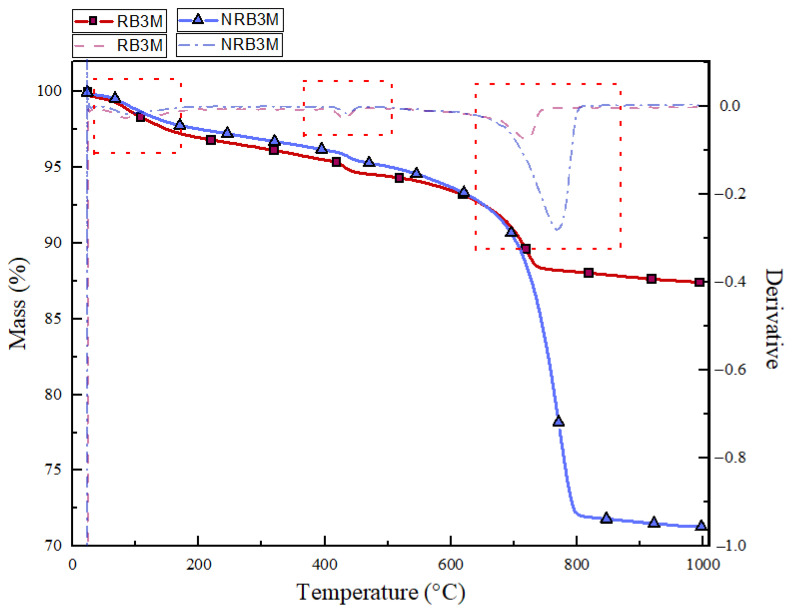
TGA/DTG analysis graph for the 3-month-exposed NRB and RB sample.

**Figure 8:**
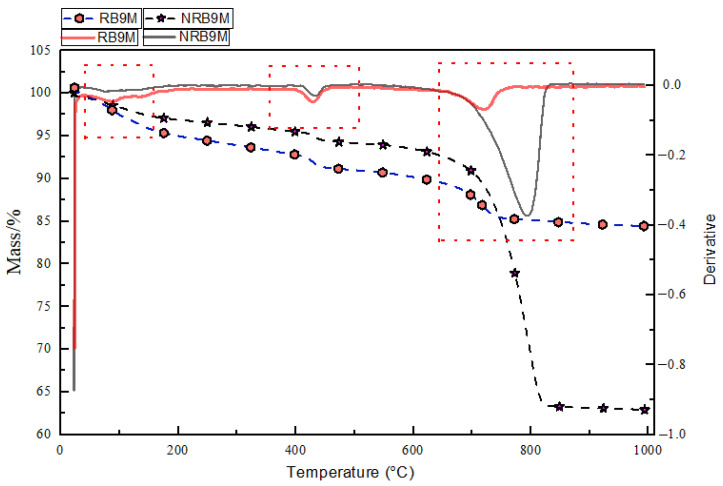
TGA/DTG analysis graph for the 9-month-exposed NRB and RB sample.

The Thermogravimetric (TGA) and derivative thermogravimetric (DTG) curves for ASR-contaminated samples are demonstrated in Figure 7 and Figure 8. Figure 7 and Figure 8 include the TGA sintering and DTG results for 3- and 9-month-exposed samples, highlighting the characteristic mass loss. By further comparing with previous research, the C-N-S-H, C-K-S-H were compared in 100 to 200 °C, dihydroxylation of Ca(OH)_2_ in 450 to 600 °C, and decarbonization of CaCO_3_ in the 600–800 °C range [57,58,66]. Figure 7 demonstrates the decomposition of 3-month-exposed NRB and RB samples. In the low-temperature range from 25 °C to 200 °C, NRB3M shows a continuous but gradual decline in mass. This loss is attributed primarily to the evaporation of physically bound water and dehydration of low-temperature hydration products, including C–S–H gel and potentially minor alkali–silica reaction (ASR) gels. Compared to RB3M (reactive beam), between 100 and 200 °C, the RB3M curve exhibits a slightly larger mass loss and a broader, more negative DTG shoulder than NRB3M, consistent with the release of physically bound and gel water from C-S-H and early ASR gels (C–N–S–H/C–K–S–H) [52].

Furthermore, the temperature range of 450–600 °C corresponds to the decomposition of portlandite (Ca (OH)_2_). The DTG profile for NRB3M displays a pronounced valley in this temperature range, indicating a substantial content of unreacted calcium hydroxide. This observation is significant as it implies minimal Ca(OH)_2_ participation in forming a calcium-rich ASR gel [57]. In the 450–600 °C interval, where portlandite (Ca(OH)_2_) dehydroxylates, the NRB3M DTG minimum is deeper and sharper, whereas RB3M shows a muted peak. This difference indicates the consumption of Ca(OH)_2_ in the reactive specimen as calcium is scavenged to form ASR gels and secondary C-S-H, leaving less free portlandite to decompose. From 600 to 800 °C, the NRB3M curve displays a pronounced DTG valley accompanied by a steep mass drop (calcite decarbonation), while RB3M shows a smaller, broader response; the weaker decarbonation in RB3M is consistent with its lower prior portlandite content (less available to carbonate) and with progressive gel filling that limits later carbonation [32,67]. Overall comparison of the stronger low-temperature dehydration, reduced portlandite peak, and subdued decarbonation peak in RB3M confirms early ASR activity: water-rich gel formation, Ca(OH)_2_ consumption, and a microstructure already shifting toward gel-rich, partially pore-blocking products, while the non-reactive concrete retains a more conventional hydrate assemblage with higher C-H composite and carbonate contents.

Figure 8 shows that the reactive specimen after 9-month exposure has demonstrated a distinct ASR gel presence. In the temperature range of 25–200 °C, RB9M exhibits a total mass loss of approximately 4–5%, which is slightly greater than that of NRB9M according to previous research by Ma et al. The ASR gel generally appears in the 100–200 °C range [52]. This region reflects the evaporation of physically bound water and gel water associated with hydration products such as C-S-H and ASR gel [68]. The elevated water loss at this stage indicates substantial ASR gel formation, as gels retain a higher amount of pore solution than crystalline hydration phases. Moreover, in the 450–600 °C range, which corresponds to the thermal decomposition of portlandite (Ca (OH)_2_) [68], the DTG curve for RB9M is significantly shallower and broader than that of NRB9M. The reduced intensity of this peak suggests a lower residual Ca (OH)_2_ content in the RB9M matrix. Quantitatively, the mass loss in this region for RB9M is less than 2%, compared to approximately 3–4% in NRB9M, indicating a relative reduction in Ca (OH)_2_ of over 30% [29]. Between 600 and 800 °C (calcite decarbonation) [53], NRB9M displays a pronounced DTG valley with a steep mass drop, while RB9M shows a smaller, broader response. The lower DTG curve illustrates less calcium carbonate due to the presence of ASR gel and the reduction of calcium hydrate bonds. The 600–800 °C range demonstrates a decrease in calcium carbonate, likely due to the presence of ASR gel and the reduction of calcium hydrate bonds [29]. Moreover, the blockage of carbon dioxide due to ASR gel also shows low carbonate reaction. The low decarbonization of calcium carbonate results in reduced calcium hydroxide formation and carbonation reactions due to ASR consumption of calcium hydroxide. Overall, the stronger dehydration in the 100–200 °C range, the weakened peak at the 450–600 °C range, and the subdued decarbonation in the 600–800 °C range provide evidence of ASR gel production after 9 months of exposure.

### 4.3. Resistance Variation in the Non-Reactive and Reactive Samples

**Figure 9:**
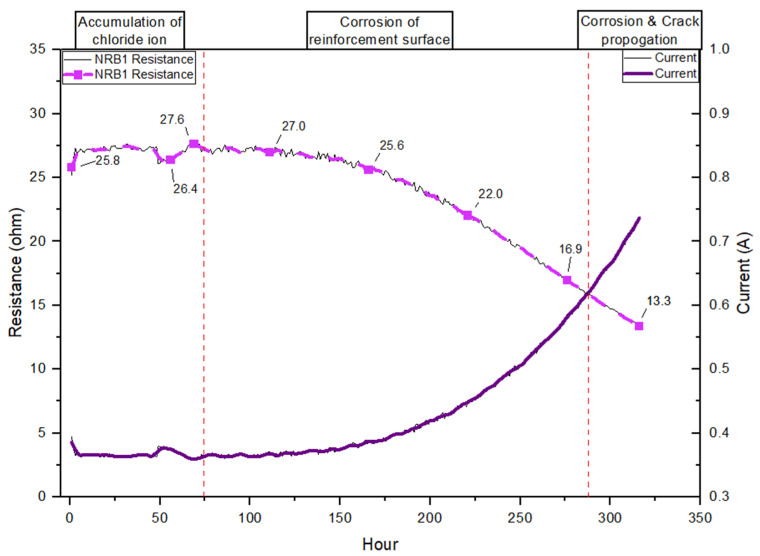
Resistance performance of chloride-corrosion exposure for the NRB sample after ASR exposure.

Figure 9 illustrates the resistance and current profile of a non-reactive specimen under the design chloride-induced corrosion exposure conditions with a constant 10 V DC after 9 months of ASR exposure. The resistance performance is classified into three stages: the accumulation of chloride ions stage, the corrosion of the reinforcement surface stage, and the corrosion and crack propagation stage. This performance is partially consistent with previous research observations of concrete electrical resistivity in chloride-induced corrosion [69]. At the initial stage of the curve, resistance is markedly high (25.8 Ω to a maximum of 27.6 Ω), corresponding to a low current flow. The initial stage shows that samples are minimally saturated, and limited ionic conduction has started. The non-reactive sample at this stage remains highly resistive. As the applied voltage continues to drive the system, a sharp increase in current is observed, accompanied by a rapid decline in resistance. As Figure 9 presents, the resistance dropped from a maximum of 27.6 Ω to stage two’s lowest point, around 15 Ω. This phase indicates a transitional period where the pore structure begins to support increased ionic kinetics. The resistance decreases further, indicating that chloride ions have penetrated the concrete cover. The enhanced ionic movement facilitates electrical conduction, thereby reducing resistance. This behaviour aligns with electrochemical theory, where increasing ionic availability within the pore solution reduces the bulk resistivity of the concrete [8,70,71].

In the third stage, the graph indicates that the resistance trend flattens, approaching a minimum value even as the current increases. This plateauing behaviour reflects the corrosion propagation phase, where the steel is actively corroding. Corrosion products, such as Fe (OH)_2_ and Fe (OH)_3_, form, and significant cracking along the reinforced direction is observed on the concrete surface due to the leaching of corrosion products. The resistance performance of this study is consistent with reported degradation mechanisms in long-term exposure cases and laboratory studies using accelerated methods [22,46,48].

**Figure 10:**
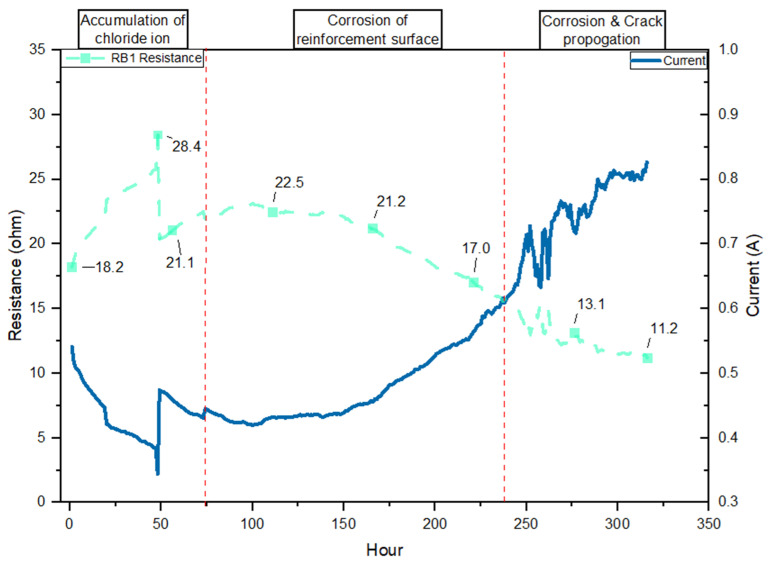
Resistance performance of chloride-corrosion exposure for the RB1 sample after ASR exposure.

**Figure 11:**
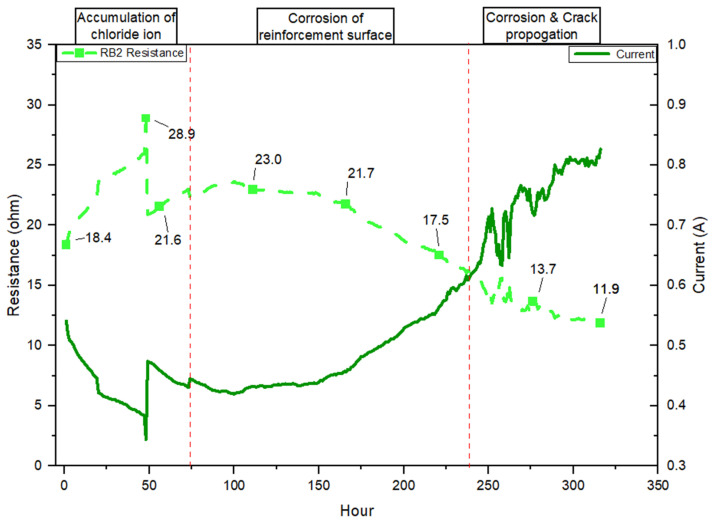
Resistance performance of chloride-corrosion exposure for the RB2 sample after ASR exposure.

Based on the resistance versus current graphs for RB01 and RB02 under constant 10 V (Figure 10 and Figure 11), the corrosion process is divided into three distinct phases: chloride ion accumulation, corrosion initiation on the reinforcement surface, and corrosion and crack propagation. These phases, demarcated by red dashed lines, correspond to electrochemical and mechanical transitions described in both theoretical corrosion models and the experimental literature. Regarding Figure 10 and Figure 11, the initial phase resistance of RB01 and RB02 increases from 18.2 Ω and 18.4 Ω to 28.4 Ω and 28.9 Ω, respectively (Table 5). This stage represents the early ingress of chloride ions into the concrete pore structure. The performance of resistance and current is consistent with the chloride penetration mechanism described in Apostolopoulos et al.’s research on the CIC-impacted performance of reinforced concrete [48]. According to Brenna et al. [72], in the absence of de-passivation, the steel remains electrochemically passive, and the potential gradient across the concrete remains stable, maintaining high resistance values.

The second stage, presented in Figure 10 and Figure 11, shows a distinguished drop in resistance (RB01: 28.4 to 17.0 Ω, RB02: 28.9 to 17.5 Ω (Table 5)) with a corresponding increase in current (from 0.33 A to 0.57 A). The resistance drop signifies that the chloride concentration at the steel surface has exceeded the critical threshold (for GP concrete, this is considered to be 0.4% by cement mass [8,73]). Previous research by Andrade et al. has further supported the stage two resistance performance [74]. In the second stage, the steel transitions from a passive to an active state, and ferrous ions begin to form at anodic sites. These reactions are facilitated by the increased ionic conductivity in the now more saturated concrete matrix. The high correlation between the observed drop in resistance and the rise in current confirms that this stage reflects the onset of corrosion reactions [46].

After 250 h of exposure, the resistance in both RB01 and RB02 declines further to values near 11.2 and 11.9 Ω, respectively, while the current rises steadily beyond 0.8 A. The distinguished shift reflects the corrosion propagation stage, where the electrochemical activity intensifies, and secondary effects such as internal rust formation, expansive stress, and microcracking occur within the concrete matrix. Research by Cao et al. and Feng et al. indicates that this stage corresponds to the propagation stage of CIC, characterized by accelerating localized corrosion and expanding rust layers [24,49]. The rapid current fluctuations in both RB01 and RB02 during this phase indicate that cracks resulting from CIC formation and rust expansion are actively altering the internal structure and electrochemical interface.

Table 4 summarizes the resistance performance comparison between the non-reactive beam (NRB) and the reactive beams (RB01 and RB02), which distinguishes apparent variations in initial conditions, degradation, and corrosion behaviour. The NRB sample at the initial stage reflects the concrete microstructure that is maintained after ASR exposure and exhibits low early permeability. The higher residual resistance in the latest stage indicates the structure restrained electrochemical activity due to the absence of ASR. In contrast, RB01 and RB02 both start with notably lower initial resistance values, approximately 18.2 and 18.4 ohms, respectively. This lower baseline is attributed to internal microcracking and increased pore connectivity resulting from ASR. These reactive beams exhibit a rapid increase in resistance during the early exposure period (up to ~70 h), followed by a sharp decline that signals the initiation of early corrosion. Both RB01 and RB02 experience corrosion onset at approximately 70 h, far earlier than NRB, as chloride ions penetrate more freely through ASR-damaged pathways. Resistance continues to drop significantly through the propagation phase, with final values reaching around 11.2 ohms for RB01 and 11.9 ohms for RB02.

### 4.4. Non-Steady-State-Migration Coefficient (D_nssm_) Analysis

**Figure 12:**
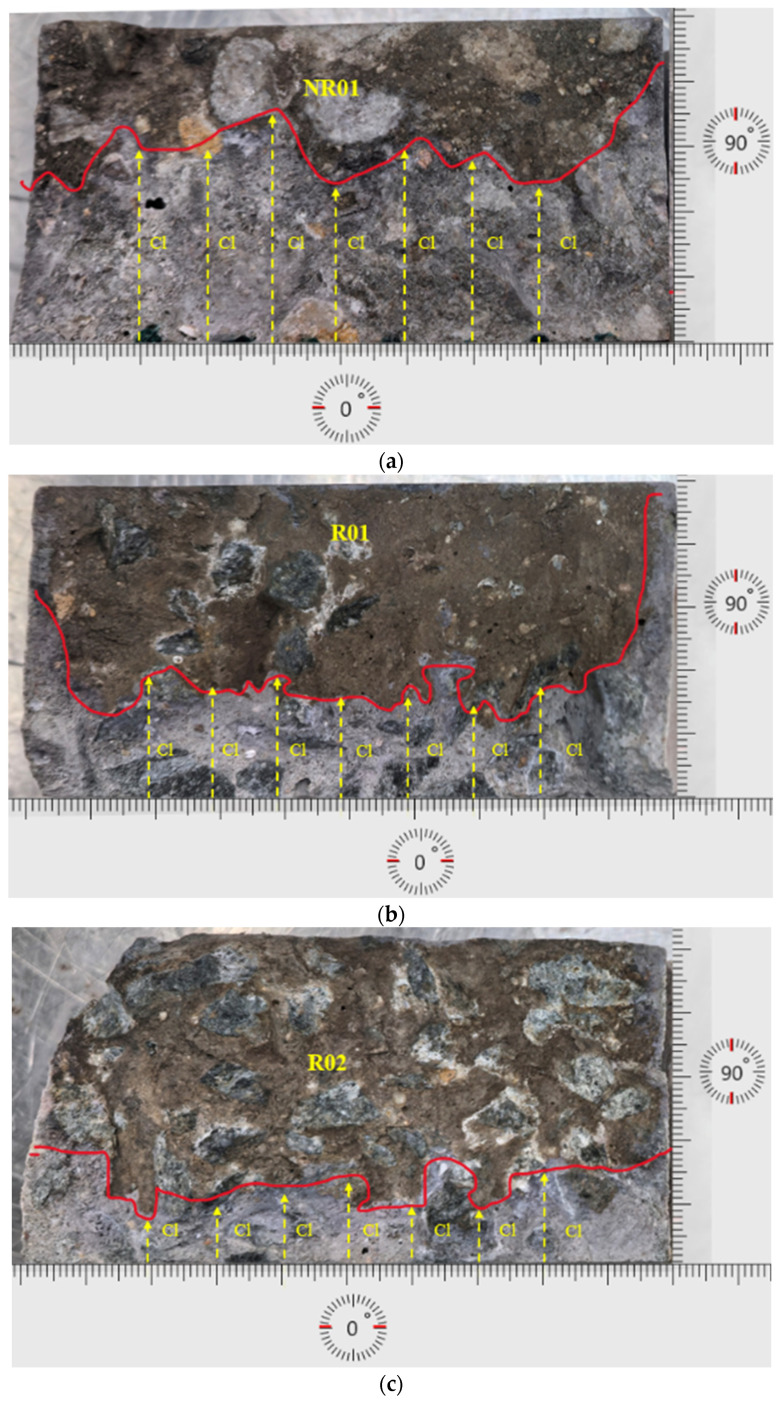
(**a**–**c**): 1-year ASR-exposed NR and R sample chloride penetration depth measurement after NT Build 492.

**Figure 13:**
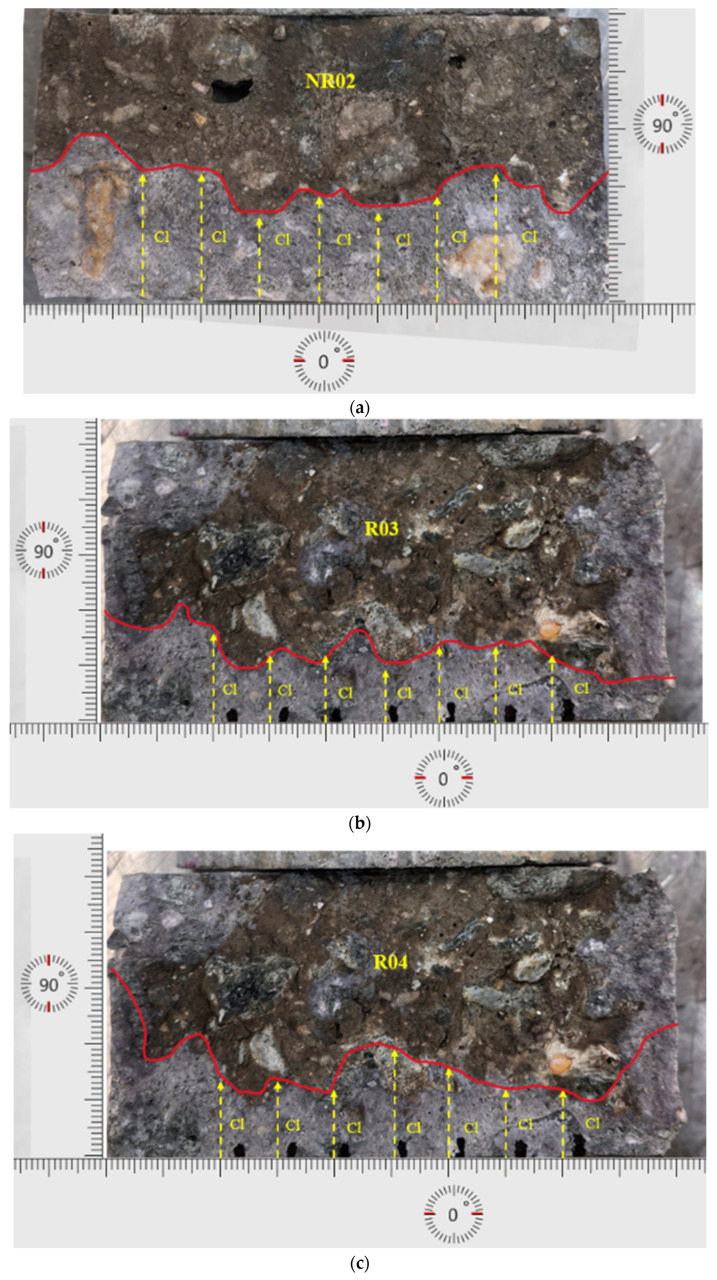
(**a**–**c**): 2-year ASR-exposed NR and R sample chloride penetration depth measurement after NT Build 492.

The NT Build 492 results are accompanied by a visual analysis of the concrete samples. The test samples are after 1 year and 2 years of exposure to a high-alkali concentration solution for ASR performance evaluation. Group one samples are after 1 year of exposure. The silver nitrogen indicator results of the samples are shown in Figure 12a–c. Group two samples are shown in Figure 13a–c. Moreover, Table 5 highlights significant differences in chloride migration characteristics. Specifically, the non-reactive sample NR01 has a notably higher non-steady-state migration coefficient (D_nssm_ = 15.09 × 10^−12^ m^2^/s), reflecting higher permeability and thus easier chloride ingress. The visual inspection of NR01 confirms relatively smooth and consistent deterioration patterns, characterized by fewer and less pronounced microcracks, indicating that chloride ions penetrate mainly through inherent porosity without substantial obstruction.

Figure 13 shows samples after two years of ASR exposure (NR02, R03, and R04); this set of specimens further reveals the interruption of ASR to chloride-induced corrosion. As Table 6 shows, the non-reactive sample NR02 exhibits a relatively high non-steady-state migration coefficient (D_nssm_ = 10.92 × 10^−12^ m^2^/s), indicating a considerable level of chloride permeability. Visually, NR02 exhibits moderate surface deterioration and limited crack development, characterized by a relatively smooth boundary line and uniform chloride ingress paths. In contrast, the reactive samples R03 (D_nssm_ = 6.93 × 10^−12^ m^2^/s) and R04 (D_nssm_ = 4.78 × 10^−12^ m^2^/s) exhibit substantially lower chloride migration coefficients, indicating significantly enhanced resistance to chloride penetration.

Figure 13a–c demonstrate extensive and irregular cracking, which typically implies higher permeability. However, these lower D_nssm_ values strongly suggest that the extensive ASR-induced micro- and macro-cracks in reactive samples have been partially filled or blocked by expansive ASR gels. These gels alter the pore network, thereby effectively mitigating chloride diffusion pathways. Among all the samples, R04 notably shows the lowest coefficient, reflecting the most significant resistance to chloride penetration despite having the most visibly severe surface deterioration. This observation highlights the crucial role of ASR-gel formation and its impact on microstructural refinement within the concrete, demonstrating a pronounced ability to inhibit chloride ingress, even in severely cracked conditions.

To account for the inherent variability of the NT BUILD 492 chloride migration test and ensure the reliability of the observed differences in chloride transport, all Dnssm values reported in this study are based on triplicate disc specimens extracted from each beam. The average Dnssm values are presented alongside their respective standard deviations in Table 6. For the non-reactive control specimens (NR01 and NR02), the Dnssm values after one and two years of exposure were 15.09 ± 1.28 × 10^−12^ m^2^/s and 10.92 ± 1.04 × 10^−12^ m^2^/s, respectively. In contrast, reactive specimens R01–R04 exhibited significantly lower Dnssm values ranging from 7.03 ± 0.51 × 10^−12^ m^2^/s to 4.78 ± 0.48 × 10^−12^ m^2^/s, reflecting relative reductions of approximately 47–56% compared to their non-reactive counterparts. The standard deviation of each group remained within 6–10% of the mean, consistent with the expected reproducibility of NT BUILD 492 testing. These results confirm that the observed reductions in chloride migration for ASR-contaminated specimens are not only systematic but also statistically meaningful, exceeding the expected experimental scatter.

## 5. Discussion

The SEM images clearly show the formation and propagation of ASR gel-filled cracks extending from reactive aggregates into the surrounding cement paste, consistent with ASR deterioration mechanisms described in previous ASR research [50]. EDS analysis identifies significant compositional shifts toward silica-rich, alkali-containing gels (C–N–S–H and C–K–S–H), highlighting their expansive nature [52]. Additionally, thermogravimetric analysis (TGA) reveals an increased consumption of portlandite (Ca(OH)_2_) and the formation of ASR-related gels, confirming substantial chemical alteration within the reactive matrix [56]. TGA further illustrates that reactive samples exhibit a notable reduction in calcium hydroxide (~30%) after nine months of exposure [51,52,53]. The cracks generated by expansive ASR gel result in lower initial electrical resistance, which is observed in resistance performance analysis. As Figure 9, Figure 10 and Figure 11 present, the lower initial electrical resistance observed in ASR-contaminated (RB) samples relative to NRB samples primarily results from the internal microstructural changes induced by ASR [12]. Regarding Trejo et al.’s research on the influence of ASR on chloride-induced corrosion [11], the ASR-induced cracks on the concrete structure’s surface increase the exposure region and enhance chloride ion accumulation. Meanwhile, the surface cracks due to ASR deteriorated concrete cover, which enhanced permeability and ionic mobility within the concrete matrix [49].

With further resistance performance, the magnitude of reactive samples showing a decrease in corrosion initiation is comparatively modest. The reasoning behind these less pronounced resistance drops lies in the fact that the initial microstructural deterioration from ASR has already substantially reduced the concrete’s resistance baseline [60]. Meanwhile, according to previous research on combined deterioration, the propagation of ASR gel in microcrack blocks prevents chloride penetration [28,31,75]. In contrast, the NRB samples, initially possessing a dense and relatively intact microstructure, maintain high electrical resistivity at the outset. Once the critical chloride threshold is surpassed, corrosion reactions rapidly degrade this dense matrix, dramatically altering the pore structure and leading to significant reductions in resistivity [8,12]. Apostolopoulos et al. (2013) provided evidence supporting this concept, demonstrating that a higher degree of initial microstructural integrity corresponds to a more pronounced decline in resistance [48].

The results obtained from impressed-current accelerated corrosion tests should be interpreted with consideration of their practical limitations. Although the method effectively distinguishes the corrosion behaviour of ASR-contaminated and control specimens, the absolute values of corrosion current, resistance loss, or time to visible damage may not directly translate to service-life predictions under natural exposure. The accelerated nature of the test may mask some synergistic effects of environmental parameters such as fluctuating moisture, temperature cycles, or gradual chloride ingress. Moreover, the corrosion morphology observed in this study—characterized by relatively rapid current-induced bar-parallel cracking—may differ from pit-initiated or diffuse corrosion patterns typical of marine environments. These factors underscore the importance of complementing accelerated electrochemical methods with field exposure studies or natural weathering trials to validate long-term durability models. Nevertheless, within the constraints of the test method, the comparative trends observed—namely the earlier resistance decline and chloride transport differences in ASR-affected beams—remain robust and instructive for understanding coupled deterioration mechanisms.

The steel condition evaluation undertaken in this study was intentionally limited to qualitative visual inspection and photographic documentation, and does not include detailed quantification of mass loss, pit geometry, or corrosion penetration profiles. As a result, the presented data does not allow for precise estimation of cross-sectional loss or direct calibration of corrosion rate models. This represents a limitation when interpreting the absolute severity of corrosion, and the findings should therefore be viewed primarily in a comparative sense, i.e., contrasting the corrosion patterns of reactive and non-reactive beams under identical electrochemical and exposure conditions. Nevertheless, the observed differences in surface rust distribution and bar-parallel cracking are consistent with the measured resistance changes and chloride transport behaviour, lending additional support to the conclusion that prior ASR damage modifies the spatial development of corrosion.

NT build 492 tests further identified the resistance variation between NRB and RB samples. Samples after NT build 492 tests revealed further elucidation of the chloride migration behaviour within ASR-affected concrete. Despite the early reduction in resistance and accelerated corrosion initiation, for instance, after one year of ASR exposure, reactive samples R01 and R02 exhibit D_nssm_ values of 7.03 × 10^−12^ m^2^/s and 8.02 × 10^−12^ m^2^/s, respectively, compared to 15.09 × 10^−12^ m^2^/s for the non-reactive sample NR01. Similarly, after two years, reactive samples (R03: 6.93 × 10^−12^ m^2^/s, R04: 4.78 × 10^−12^ m^2^/s) exhibit even lower migration coefficients compared to the non-reactive sample NR02 (10.92 × 10^−12^ m^2^/s). This reduction in chloride migration coefficients, despite visible macro-cracking, suggests that ASR gels formed in microcracks act as an internal sealant. ASR gels, identified through SEM-EDS analyses as C–N–S–H and C–K–S–H phases, exhibit expansive and hygroscopic characteristics that partially block existing cracks and pores over extended periods [52,53]. Such counterintuitive results indicate that although initially reactive samples show increased susceptibility, the formation of expansive ASR gels progressively refines internal microstructures, partially sealing the ASR-induced cracks and reducing chloride migration paths [31,76].

Macroscopic visual inspections of concrete surfaces reveal extensive and irregular cracking in reactive samples, indicative of substantial ASR damage. However, NT Build 492 results paradoxically show reduced chloride penetration depth compared to less-cracked non-reactive samples (Figure 12 and Figure 13). The severe surface cracking contrasts with the refined internal microstructure achieved through gel deposition, revealing a disconnect between visual damage assessments and actual chloride resistance performance. Thus, macroscopic visual assessment alone would misleadingly suggest higher chloride susceptibility, whereas microscopic and NT Build 492 analyses provide a more accurate assessment of actual chloride resistance. This complex macro-micro interplay emphasizes the importance of comprehensive multi-scale analysis when evaluating structural durability.

## 6. Conclusions and Recommendations

1. The study investigated the combined effects of the alkali–silica reaction (ASR) and chloride-induced corrosion (CIC) on reinforced concrete beams made with non-reactive blue metal and reactive dacite aggregates under a high-alkali, alkali-equilibrium immersion regime followed by impressed-current corrosion and NT BUILD 492 testing.
2. After long-term ASR exposure, reactive beams exhibited extensive surface map cracking with representative crack widths up to 1 mm, whereas non-reactive beams remained comparatively undamaged at the surface.
3. Despite more severe visible cracking, reactive beams showed consistently lower non-steady-state chloride migration coefficients (Dnssm) than non-reactive beams, with reductions of approximately 47–53% after one year and 37–56% after two years, supported by statistical analysis of test variability.
4. Electrical resistance monitoring indicated that ASR-contaminated beams tended to exhibit earlier resistance decline (suggesting facilitated corrosion initiation) but not necessarily greater resistance loss during the later propagation stage.
5. SEM–EDS and TGA results confirmed extensive ASR product formation and matrix alteration, supporting a time-dependent, two-stage mechanism in which early microcracking promotes transport, while subsequent gel accumulation and local densification partially obstruct transport paths and reduce effective chloride migration.
6. Limitations include the lack of detailed monitoring of alkali leaching, qualitative rather than quantitative steel mass-loss data, and the potential non-representativeness of impressed-current corrosion morphologies relative to natural field conditions.

Overall, this study offers vital insights into the nuanced, time-dependent relationship between ASR-induced microstructural alterations and chloride-induced corrosion, which is essential for accurate durability assessments and informed infrastructure management in chloride-rich environments. For future research, the pH value changes before, after, and after CIC exposure need further clarification. Meanwhile, the bonding area compromise required microstructure investigation. Both future research suggestions are critical to developing an understanding of the combined deterioration relation and interruption.

## Figures and Tables

**Table 1:** Chemical content of GP cement, Boral.

Chemical Compositions (%)	GP
SiO_2_	14.15
P2O_5_	0.17
SO_3_	1.83
CaO	49.24
K_2_O	0.74
**Alkali chemical**	**Content**
K_2_O	0.45%
Na_2_O	0.12%
**Sodium oxide equivalent**	**Content**
Na_2_Oeq	0.42%

**Table 2:** Concrete mix design.

Concrete Mix Proportions	Reactive	Non-Reactive
Water–binder ratio	0.4	0.4
Mixing water	176	176
Cement kg/m^3^	440	440
Coarse aggregate 20 mm kg/m^3^	794	794
Coarse aggregate 10 mm kg/m^3^	340	340
Sand kg/m^3^	610	610
Alkali boost	1.1%	1.1%

**Table 3:** RC sample arrangement.

Beam Sample	Purpose
NRB 1, 2; RB 1, 2	ASR deterioration, then CIC exposure
Cylinder samples	3, 6, 9 months SEM-EDS, XRD

**Table 4:** SEM-EDS chemical detection points summary.

Point	Location/Feature	Si wt.%	Ca wt.%	Na + K wt.%	Cl wt.%
**P1**	Reaction rim at aggregate–cement paste boundary (rim densification)	5.2	20.7	1.2	0.68
**P2**	Near aggregate–gel interface	21.2	14	1.7	0.12
**P3**	ASR gel in a microcrack	16.5	12.7	2	-
**P4**	Cement paste	7.8	24.4		1.43
**P5**	Alkali-leached gel/silica-rich zone in a crack	30.3	2.5	1	0.14
**P6**	Altered gel adjacent to the cement matrix	10.2	20.9	3.1	1.12
**P7**	Crack-filling gel (active zone)	38.8	5.5	6.5	-
**P8**	Crack-filling gel (active zone)	38.8	5.5	6.5	-

**Table 5:** Summary of transitions of resistance and current variation.

Phase	Time (Hours)	Resistance Trend (Ω)	Current Trend (A)	Electrochemical Interpretation
**Chloride Accumulation**	0–70	18 → 28.4/28.9	constant (~0.3 A)	Chloride diffusing; passive steel
**Corrosion Initiation**	70–240	28.4 → 17.0	0.3 → 0.55 A	Passive film breaks down; Fe^2+^ and OH^−^ form
**Corrosion and Crack Propagation**	240–330	17.0 → 11.2/11.9	0.55 → 0.8 + A	Rust expands; cracks grow; corrosion accelerates

**Table 6:** Non-steady-state migration coefficient (D_nssm_) of ASR-exposed samples.

	Mix	Voltage	D_nssm_
**1-year ASR-exposed**	NR01	30	15.09
	R01	30	7.03
	R02	30	8.02
**2-year ASR-exposed**	NR02	30	10.92
	R03	30	6.93
	R04	30	4.78

## Data Availability

The original contributions presented in this study are included in the article. Further inquiries can be directed to the corresponding authors.
